# Identifying food deserts and swamps based on relative healthy food access: a spatio-temporal Bayesian approach

**DOI:** 10.1186/s12942-015-0030-8

**Published:** 2015-12-30

**Authors:** Hui Luan, Jane Law, Matthew Quick

**Affiliations:** Faculty of Environment, School of Planning, University of Waterloo, 200 University Avenue West, Waterloo, ON Canada; Faculty of Applied Health Sciences, School of Public Health and Health System, University of Waterloo, 200 University Avenue West, Waterloo, ON Canada

## Abstract

**Background:**

Obesity and other adverse health outcomes are influenced by individual- and neighbourhood-scale risk factors, including the food environment. At the small-area scale, past research has analysed spatial patterns of food environments for one time period, overlooking how food environments change over time. Further, past research has infrequently analysed relative healthy food access (RHFA), a measure that is more representative of food purchasing and consumption behaviours than absolute outlet density.

**Methods:**

This research applies a Bayesian hierarchical model to analyse the spatio-temporal patterns of RHFA in the Region of Waterloo, Canada, from 2011 to 2014 at the small-area level. RHFA is calculated as the proportion of healthy food outlets (healthy outlets/healthy + unhealthy outlets) within 4-km from each small-area. This model measures spatial autocorrelation of RHFA, temporal trend of RHFA for the study region, and spatio-temporal trends of RHFA for small-areas.

**Results:**

For the study region, a significant decreasing trend in RHFA is observed (-0.024), suggesting that food swamps have become more prevalent during the study period. For small-areas, significant decreasing temporal trends in RHFA were observed for all small-areas. Specific small-areas located in south Waterloo, north Kitchener, and southeast Cambridge exhibited the steepest decreasing spatio-temporal trends and are classified as spatio-temporal food swamps.

**Conclusions:**

This research demonstrates a Bayesian spatio-temporal modelling approach to analyse RHFA at the small-area scale. Results suggest that food swamps are more prevalent than food deserts in the Region of Waterloo. Analysing spatio-temporal trends of RHFA improves understanding of local food environment, highlighting specific small-areas where policies should be targeted to increase RHFA and reduce risk factors of adverse health outcomes such as obesity.

## Background

Past research has demonstrated that the food environment is an important factor in health outcomes. Several studies have shown that residents with higher access to healthy foods have healthier diets [1], lower risk of overweight/obesity [2], and lower risk of high blood pressure [3]. Obesity, in particular, is a major risk factor for chronic diseases including heart diseases, stroke, and diabetes [4].

Acknowledging the role of healthy food access in shaping food consumption and related health outcomes, policymakers have prioritized increasing healthy food access. In Canada, for example, the Ontario Professional Planners Institute has issued a call for action on planning for healthy food systems and engaging planners with food relevant issues [5]. Furthermore, the municipalities of Vancouver [6] and Toronto [7] have developed local programs to increase healthy food access by establishing healthy corner stores that sell fresh produce and instituting mobile grocery stores.

### Measuring the food environment

Various measures have been developed for assessing the food environment and have been summarized [8–11] and compared [12, 13] in extant literature. While these measures can be categorized based on a number of different criteria (e.g., community or consumer nutrition environment^1^ [10]), one important distinction is between absolute and relative measures.

The absolute and relative measures capture different aspects of the food environment [12]. Absolute metrics (e.g., the density of supermarkets within a census tract) measure access to one type of food outlet whereas relative metrics assess the relative accessibility of two types of food outlets, including healthy and unhealthy [14]. Recent research has demonstrated that relative healthy food access (RHFA), as measured by the percentage of healthy food outlets (=healthy outlets/healthy + unhealthy outlets), better represents food purchasing and consumption behaviours [15, 16] compared to absolute densities of healthy food outlets. This may be because RHFA measures the balance between healthy and unhealthy food outlets while absolute measures assess only a portion of the total food environment. While analysed in past research, relative measures has been shown to provide more consistent and expected associations with health outcomes. In a meta-analysis of 61 studies, Zenk et al. [17] observed four studies that employ relative food environment measures and all of these studies had consistent and expected findings (e.g., higher RHFA linked to lower odds of obesity), whereas mixed findings were identified in studies using absolute food environment measures. Relative measures also have methodological advantages since incorporating both absolute measures of healthy and unhealthy food outlets in regression models could lead to multi-collinearity as these two measures are usually positively correlated [16].

Capturing both healthy and unhealthy food outlets in one measure allows for a more comprehensive analysis of different dimensions of the food environment [18] and enables conceptualizing food deserts and food swamps on a continuous scale. Food deserts are areas lacking access to nutritious and affordable food (i.e., 0 % RHFA) and food swamps are areas that with relatively few healthy options (i.e., small RHFA) [19] or where “large relative amounts of energy-dense snack foods, inundate healthy food options” [20]. The modified Retail Food Environment Index (mRFEI) is a relative measure of the food environment that can represent both food deserts and food swamps^2^ where a value equal to zero characterizes a food desert while a small value greater than zero characterizes a food swamp. Food deserts have been extensively investigated in past research, however recent research indicates food swamps may be more prevalent in countries including Canada [20–22].

### Temporal variation in the food environment

Previous research has indicated that changes in the numbers and types of retail food outlets may lead to changes in food purchasing and consumption behaviours [23], however little research has analysed temporal changes in healthy food access, especially RHFA.

Temporal food access can be considered from supply (retail) and demand (consumer) sides. From the supply side, variations in temporal food access occur across years (e.g., new food outlets opening), seasons (e.g., farmers’ markets), and weekdays (e.g., opening hours of food outlets) [23–27]. For example, Filomena et al. [23] investigated annual changes of the food environment in Brooklyn, New York between 2007 and 2011 and observed that changes in absolute healthy food outlets varied between neighbourhoods based on income and ethnic composition, where low income and predominately non-white neighbourhoods experienced higher variations in healthy food access. Widener et al. [25] found that poorer neighbourhoods have better spatial access to healthy foods in summer than in winter because of seasonal farmers’ markets. Also analysing food environments at the seasonal scale, Lamichhane et al. [28] explored associations between absolute densities of supermarkets, convenience stores and socio-demographic characteristics. Positive associations were observed between the number of both types of food stores and neighbourhood poverty. Two recent studies from Chen and Clark [26, 27] suggested that socio-economically marginalized neighbourhoods have limited temporal access, rather than spatial access, to healthy food outlets due to limited daily opening hours of green retailers. Therefore, interventions such as extending opening hours of green retailers were recommended to reduce healthy food access disparities, complementing conventional interventions (e.g., building new healthy food outlets).

From the demand side, temporal food access is generally measured for individuals because it is largely determined by consumer time availability (e.g., people working non-conventional hours may be constrained by food outlet operating hours [26]). In this case, the space–time prism has been used to quantify food accessibility, incorporating individual mobility and time budgets [29, 30]. Findings from these studies identify which population rather than which areas have greater or less access to healthy foods. Temporal variations in transportation service (especially public transit) that link supply and demand sides also influence healthy food access. For example, Farber et al. [31] found that supermarket accessibility varied for public transit-dependent residents across the day in Cincinnati due to daily fluctuations in transit availability.

This study analyses annual spatio-temporal variations of RHFA at the small-area scale for the Region of Waterloo, from 2011 to 2014, complementing past research that analyses only spatial variations and absolute healthy food access. RHFA at a small temporal scale (e.g., annual) merits attention given that changes in the number and type of food outlets are slow and it probably takes a long time for the food environment to manifest its health effects [32]. Specifically, this study has three objectives: (1) to estimate temporal trend in RHFA for the study region (regional trend), (2) to identify spatio-temporal RHFA trends at the small-area scale (local trends), and (3) to highlight spatio-temporal food swamps, or small-areas where RHFA is decreasing at a greater rate than the study region.

## Study region and data

### Study region

The Region of Waterloo, Ontario, Canada, is composed of three cities, Kitchener, Waterloo, and Cambridge, and four rural townships. It is located approximately 1 h west of Toronto, Canada’s largest city. For this study, rural townships were excluded from the analysis because retail food outlets are primarily located in urban areas. City boundaries were collected from the Region of Waterloo [33].

In total, 655 DAs with a population of 444,681 were analysed. For reference, DAs are the smallest census units that cover the entirety of Canada and are delineated according to roads and physical boundaries [34]. Average DA population density was 3234/km^2^, ranging between 2/km^2^ in a predominantly industrial DA and 16,025/km^2^ in a DA with many apartment buildings. Population data and geographic shapefiles were obtained from Statistics Canada [35].

### Food outlet data

Retail food outlet locations were extracted from a food inspection dataset containing all food outlets in the Region of Waterloo. Some misclassification of outlets was detected, which is a common challenge encountered in secondary datasets [36, 37]. Retail food outlets were re-classified based on categories from the Nutrition, Environment in Waterloo Region, Physical Activity, Transportation and Health (NEWPATH) project [38], which surveyed in-store characteristics of all food outlets (e.g., shelf-space dedicated to fruit and vegetables in a supermarket or availability of healthy eating options in a restaurant) in 2009. NEWPATH included nine categories: full-service restaurant, fast-food restaurant, bar/pub, supermarket, speciality food store, convenience store, pharmacy, superstore, and snack stand.

In practice, dichotomously categorizing food outlets as ‘healthy’ or ‘unhealthy’ is contentious because many healthy food outlets supply unhealthy food products. We followed the most common and simplest classification scheme in the literature [39]: only supermarkets/superstores are classified as healthy and only convenience stores and fast-food restaurants are classified as unhealthy. Similar approaches have been employed in recent Canadian [15, 40] and Australian [16] studies.

RHFA was calculated by dividing the number of healthy food outlets by the sum of healthy and unhealthy food outlets within a 4 km road network buffering distance from DA centroids. Food outlets that were located outside of the study region, but were inside buffers, were included. A 4 km buffering distance was chosen because RHFA within a DA is likely not representative of food purchasing behaviours, as DAs are small (average area = 0.48 km^2^) and retail food outlets are often located close to small-area borders [41]. A 4 km road network buffer approximates a 5-min driving distance, which is the primary transportation mode for employment and shopping in the study region (approximately 85 % of employed residents either drive to work or are passengers^3^). A 5-min driving distance also captures local food environments for residents using other forms of transportation, such as public transit and cycling. For reference, the longest distance from a DA centroid to the closest healthy or unhealthy food outlet is 3.53 km.

Table 1 shows the descriptive statistics for healthy and unhealthy food outlets in the study region. Between 2011 and 2014, the number of healthy food outlets slightly declined by three (4.3 %), while the number of unhealthy food outlets increased by 34 (3.6 %). As a result, RHFA for the study region decreased from 7 to 6.5 %. Notably, because the number of convenience stores decreased by 12, the increase in unhealthy food outlets is due to increasing numbers of fast-food restaurants.

**Table 1:** Descriptive statistics of retail food outlets and RHFA by year

	2011	2012	2013	2014
Healthy food outlets	70	69	68	67
Unhealthy food outlets				
Total	932	939	942	966
Convenience store	323	317	306	311
Fast-food restaurant	609	622	636	655
Total healthy and unhealthy food outlets	1002	1008	1010	1033
RHFA (%)	7	6.8	6.7	6.5

**Fig. 1:**
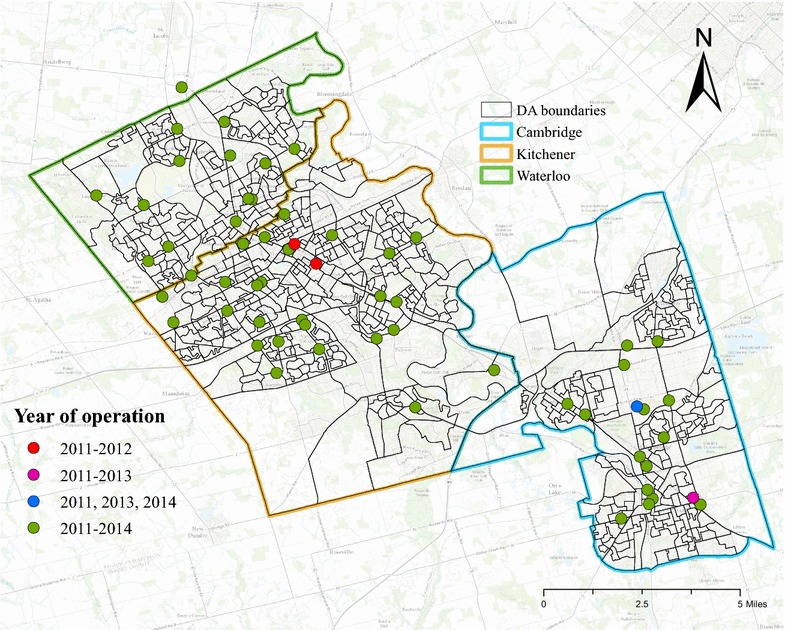
Distributions of healthy food outlets in the Region of Waterloo from 2011 to 2014

Figure 1 shows the geographic distribution of healthy food outlets in the study region from 2011 to 2014. Most healthy food outlets were operational during the 4 years (green dots), with the exception of two (red dots) in north Kitchener and one (pink dot) in south Cambridge. One healthy food outlet at middle Cambridge (blue dot) was closed in 2012, but a new one was constructed at the same site in 2013.

**Fig. 2:**
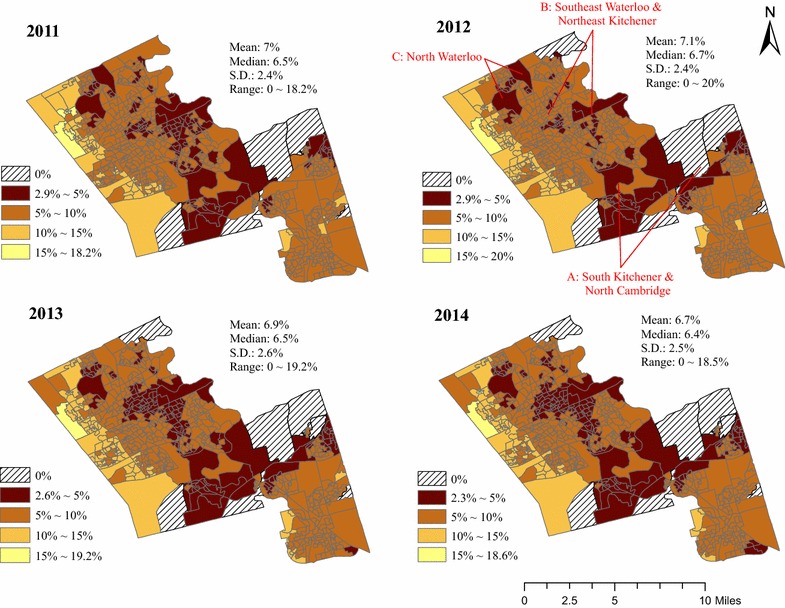
Quantile maps of RHFA from 2011 to 2014

Figure 2 maps RHFA at the DA-scale for each year. RHFA values range from 0 % in all years to 20 % in 2012. Areas that have no healthy food outlets within 4 km are highlighted with hatched lines.

While no explicit thresholds have been applied to define food swamps, we assume that they are areas where RHFA is greater than zero and less than 10 %. This is based on a recent study that demonstrated that, in areas with more than 10 % of healthy food outlets, households had higher odds of purchasing healthier foods [16]. Most DAs (~90 %) are identified as food swamps because they have low RHFA (<10 %). Some DAs have RHFA of less than 5 % for the duration of the study period and are highlighted in Fig. 2: south Kitchener and north Cambridge (Location A), southeast Waterloo and northeast Kitchener (Location B), and north Waterloo (Location C).

Notably, one DA in north Waterloo went from a food swamp in 2011 to a food desert in 2012, which was due to road network reconstructions that made supermarkets/superstores inaccessible within 4 km. While the RHFA patterns in most small-areas are similar from 2011 to 2014, RHFA fluctuations in Location B are noticeable. In 2012, RHFA increased in Location B because the number of accessible unhealthy food outlets decreased and the number of supermarkets/superstores was constant. Following closures of two supermarkets in 2013, RHFA decreased in these same areas.

## Methodology

A Bayesian hierarchical model was used to analyse the spatio-temporal trend of RHFA. This approach was adapted from Bernardinelli et al. [42] and has been widely used in spatio-temporal analysis of count data [43–45]. Bayesian approaches combine prior knowledge and observed data (i.e., accessible healthy food outlets) to estimate posterior distributions of unknown parameters (i.e., regional RHFA trend).

The spatio-temporal model consists of two levels. The first level [Model (1)] assumes that the count of healthy food outlets within 4 km of DA *i* at time *j* follows a binomial distribution, where *Y*_*ij*_ is the observed number of healthy food outlets, *T*_*ij*_ is the sum of healthy and unhealthy food outlets, and *p*_*ij*_ is the probability of a food outlet being healthy. Of note, *p*_*ij*_ can be considered as an estimated RHFA and while different than calculated RHFA, they are both representative of the risk of low RHFA. The distinction will be detailed in the discussion section.

1 $$Y_{ij} \sim Binomial\,\,(p_{ij} ,T_{ij} )$$

Using a logit link function, *p*_*ij*_ is decomposed into parameters measuring purely spatial variation, purely temporal variation, and spatio-temporal interaction at the second level [Model (2)].

2 $$\text{logit} \,\,(p_{ij} ) = \alpha + u_{i} + s_{i} + (\gamma + \delta_{i} )t_{j}$$

Purely spatial variation is represented by an intercept *α* (average RHFA for the study region), *u*_*i*_ (unstructured random effects), and *s*_*i*_ (spatially structured random effects). Random effects (*u*_*i*_and *s*_*i*_) deal with overdispersion (greater variance than expected based on a probability distribution) which occurs when modelling count data at the areal level. Sources of overdispersion in small-area studies include intra-area heterogeneity, which may be due to the presence of missing covariates or measurement errors in covariates [43, 46, 47]. The spatially structured random effects, *s*_*i*_, model the spatial autocorrelation of RHFA. Because RHFA is calculated using a buffering approach, it is likely to be spatially autocorrelated such that nearby areas exhibit similar RHFA.

In Model (2), purely temporal variation of RHFA for the study region is captured by *γ*. We assumed a linear regional trend over a four-year period considering that the opening and closure of food outlets occur infrequently over time (compared to epidemiological cases that likely vary rapidly at small-area levels over 4 years, for example) (Fig. 2). The spatio-temporal interaction term *δ*_*i*_ models local differential trends (the difference between regional trend and local trends) in RHFA after accounting for purely spatial and temporal effects. Notably, *t*_*j*_ is the centred time, calculated by subtracting the empirical mean from each time value, which has been suggested for better model convergence [48].

Model (2) can be extended to include other covariates [Model (3)]. Specifically, **X**_**i**_^**T**^ is a vector of covariates that could be included in the modelling, and **β** is a vector of corresponding coefficients. An example of covariates to be included is population density to explore the possibility that food outlets are located in highly populated areas.

3 $$\text{logit}(p_{ij} ) = \alpha + u_{i} + s_{i} + (\gamma + \delta_{i} )t_{j} + {{\textbf{X}}_{{^{\text{i}}} }}^{\text{T}} {\boldsymbol{\upbeta }}$$

The posterior probability (PP_i_) of *δ*_*i*_ being less than zero measures the strength that the local trend negatively departs from the regional trend (*γ*) [43, 44]. Spatio-temporal food swamps are small-areas that exhibit a decreasing RHFA trend and a high probability of local RHFA trend being less than regional RHFA trend. Specifically, they are areas that have a negative local trend (*γ* + *δ*_*i*_ < 0) (i.e., decreasing RHFA from 2011 to 2014) and high PP_i_ of *δ*_*i*_ less than zero (i.e., local RHFA trend strongly differs from the study region trend). Notably, spatio-temporal food swamps are not necessarily static food swamps.

We specified an improper uniform prior *U* (−∞, +∞) for the intercept *α*. Priors for spatial random effect *s*_*i*_ and spatio-temporal interaction *δ*_*i*_ were specified by the intrinsic (Gaussian) conditional autoregressive (ICAR) [49] distribution. Under the ICAR distribution, the expected mean of *s*_*i*_ and *δ*_*i*_ of the i^th^ DA is the mean of adjacent *s*_*i*_’s and *δ*_*i*_’s, respectively, where adjacency is defined as areas sharing at least one common vertex [44]. Variances of *s*_*i*_ and *δ*_*i*_ is controlled by hyperparameters^4^*σ*_*s*_^2^ and *σ*_*δ*_^2^, respectively, and is inversely proportional to the number of neighbours of the i^th^ DA.

It should be noted that there are other prior specifications for spatial parameters, for example the proper (Gaussian) conditional autoregressive distribution. ICAR is appropriate for data that exhibits high spatial autocorrelation [47, 50] and strong spatial autocorrelation of RHFA has been identified using Moran’s I^5^ (≥0.8).

A non-informative prior *Normal* (0, 1000) was given to the regional trend parameter *γ* and covariate coefficients β, respectively, while a prior of *Normal* (0, *σ*_*u*_^2^) was assigned to *u*_*i*_. Non-informative hyperpriors of *Gamma* (0.5, 0.0005) were given to the reciprocal of hyperparameters *σ*_*s*_^2^, *σ*_*u*_^2^, and *σ*_*δ*_^2^ (denoted as *τ*_*s*_, *τ*_*u*_, and *τ*_*δ*_). To determine the degree to which hyperparameter specification influenced results, we performed sensitivity analysis using three alternative priors: (1) *Gamma* (0.001, 0.001) for *τ*_*s*_, *τ*_*u*_, and *τ*_*δ*_, (2) a uniform prior U (0, 100) [44] for *σ*_*s*_, *σ*_*u*_, and *σ*_*δ*_, and (3) a half normal prior *Normal*_+∞_ (0, 10)^6^ [45, 51] for *σ*_*s*_, *σ*_*u*_, and *σ*_*δ*_.

We fitted the models using the WinBUGS software [52] with two parallel chains thinned by 10 to reduce autocorrelation. Convergence was checked by visually examining trace plots, history plots, autocorrelation plots, and Gelman-Rubin plots. Deviance Information Criterion (DIC) [53] was used to identify the model best fitting the data. The better model is the one with a smaller DIC value.

## Results

Model (2) and (3) were compared in Table 2 to identify the model that better represents the spatio-temporal variation (rather than covariates) of RHFA, which is the main goal of this study. Model (3) extended Model (2) by testing the association between RHFA and population density, one of the major driving factors of the distribution of food outlets [24, 54]. This association was found to be insignificant. A DIC difference of 1.2 (10,162.5 versus 10,163.7) does not indicate remarkable improvement of model fitting, so we selected Model (2) based on the principle of parsimony.

**Table 2:** Spatio-temporal analyses results of Model (2) and Model (3)

	Model (2)	Model (3)
Population density *β* (95 % Credible Interval)^a^	NA	0.003 (−0.015, 0.022)
Regional trend *γ* (95 % Credible Interval)	−0.024 (−0.036, −0.011)	−0.024 (−0.037, −0.011)
DIC	10,162.5	10,163.7

^a^The 95 % Credible Interval is the range in which there is a 95 % probability that the posterior mean occurs

For Model (2), convergence occurred by 10,000 iterations (thinned by 10). We ran a further 10,000 iterations for both chains to obtain 20,000 samples of the posterior distribution. Regional trend (*γ*) was negative (−0.024) and statistically significant at the 95 % credible interval, indicating a decreasing trend of RHFA at the region-scale from 2011 to 2014. The sensitivity analysis using the alternative hyperpriors discussed above suggested that results are insensitive to the selection of hyperpriors as DIC differences between Model (2) and models using the three alternative priors are only 6.3, 5.8, and 1, respectively.

**Fig. 3:**
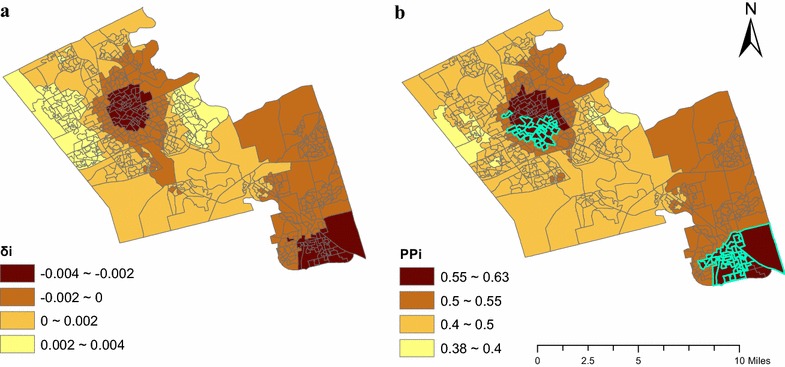
**a** Local differential trends (*δ*_*i*_) and **b** the posterior probability of a local trend less than the regional trend (PP_i_)

Figure 3a shows the area-specific differential trend, which indicates the degree to which local area-specific trends deviate from the regional trend. The map is smoothed because of the buffering approach used to calculate RHFA and the addition of spatially structured random effects.

Since the regional trend (*γ*) is −0.024 and the largest differential trend (*δ*_*i*_) is 0.004, no DAs exhibit a positive trend in RHFA (i.e., maximum trend is −0.024 + 0.004 = −0.02). A negative differential trend (*δ*_*i*_) indicates a steeper decreasing trend than the regional trend while a positive one indicates a gentler decreasing trend. Areas in the lowest quantile (−0.004 ~ −0.002) have the steepest decreasing trend and are located in south Waterloo, north Kitchener, and southeast Cambridge.

Figure 3b shows PPi, or the strength that area-specific trend negatively deviates from the regional trend. Because food outlet closures and openings are slow, PP_i_s are relatively small with the maximum 0.63. We assumed 0.55, the fifth quintile threshold of PP_i_s, to be a reasonable threshold for defining a “high” PP_i_ although higher thresholds have been used in other contexts [44, 45]. This threshold enables the top 20 % DAs to be identified as having a “high” PPi. As mentioned, areas with high PP_i_ and negative (*γ* + *δ*_*i*_) are spatio-temporal food swamps; therefore, areas in the lowest quantile (0.55 ~ 0.63; Fig. 3b) are identified as spatio-temporal food swamps given that all small areas had a decrease trend of RHFA. As shown by Fig. 3, areas with high PP_i_ coincide with areas with the steepest area-specific differential trends. This is expected as there is more evidence that these areas have a trend that negatively deviates from the regional trend. Notably, in Fig. 3b we highlight DAs that are not in the quantile with lowest RHFA (based on Fig. 2) but experienced a significant steeper decreasing trend of RHFA (more in the discussion).

## Discussion

Consistent with previous findings in the Canadian context, this paper reveals that food swamps are more prevalent than food deserts in the study region. Using a Bayesian model that accounts for spatial autocorrelation and spatio-temporal interaction, this paper also shows that food swamps are becoming more prevalent during the study period.

Past research evaluating the food environment is predominantly spatial, thus providing limited insight into how RHFA is changing over time at the local scale. For example, spatial analysis of the food environment shows that Locations A, B, and C (Fig. 2) have similar RHFA (<5 %). Results of this spatio-temporal model, however, show that there is strong evidence (high PPi) that some DAs in Location B exhibited steeper decreasing trend of RHFA (*δ*_*i*_ < −0.002) and can be categorized as spatio-temporal food swamps. Locations A and C had relatively stable RHFA and are not spatio-temporal food swamps (0 < *δ*_*i*_ < 0.002). It is noteworthy that a spatio-temporal food swamp could attribute to decreases of accessible healthy food outlets and/or increases of accessible unhealthy food outlets during the study period. For example, two DAs that are both identified as spatio-temporal food swamps in our analysis and have the same increase in fast-food restaurants; however one exhibits an increase in convenience stores (unhealthy) and the other exhibits a decrease in supermarkets/superstores (healthy).

This study has also identified areas that were not in the quantile of lowest RHFA based on only spatial and descriptive approaches, but have decreasing trends of RHFA that are steeper than the regional decreasing trend (highlighted in Fig. 3b). If the trend continues, these highlighted DAs could become new areas that have the lowest RHFA. Such temporal information cannot be quantified through visual comparison of multiple maps (Fig. 2) and can help policy makers prioritize specific areas for interventions. For instance, the spatio-temporal food swamps at south Waterloo, north Kitchener, and southeast Cambridge should be prioritized since RHFA decreases faster in these areas.

**Fig. 4:**
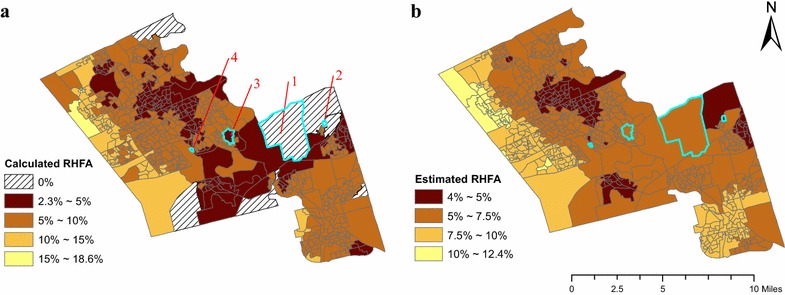
**a** RHFA in 2014 and **b** estimated RHFA in 2014 [*p*_*i*4_ in Model (2)]

As mentioned, estimated RHFA is different from calculated RHFA. Calculated RHFA is simply the number of healthy food outlets divided by the sum of healthy and unhealthy food outlets. Estimated RHFA is the probability of a food outlet being healthy [p_ij_ in Model (2)] and is based on calculated RHFA in a given DA and the average of calculated RHFA’s in adjacent areas [via the spatial random effects in Model (2)]. In this case, estimated RHFA helps to account for the realistic assumption that people could travel beyond DA or buffering zone boundaries to procure food; therefore, the RHFA value is smoothed (Fig. 4b). In contrast, calculated RHFA constraints food access within the DA or buffering zones. Two DAs with the same calculated RHFA could have varied estimated RHFA if the averages of calculated RHFA’s in their adjacent areas are different. To exemplify the difference between calculated RHFA and estimated RHFA, we selected two pairs of DAs (highlighted in Fig. 4) with the same calculated RHFA but differing estimated RHFA in 2014: one pair are food deserts (Areas 1 and 2 have calculated RHFA = 0 %) and the other pair are food swamps (Areas 3 and 4 have calculated RHFA = 4.76 %). Area 1 has a higher average of calculated RHFA’s among adjacent areas (3.58 %) compared to Area 2 (2.08 %), leading to Area 1 having a greater estimated RHFA. Similarly, Area 3 has adjacent areas with a higher average of calculated RHFA’s than Area 4, leading to Area 3 having a greater estimated RHFA. Practically, these results suggest that Area 2 is a more serious food desert than Area 1 and that Area 4 is a more serious food swamp than Area 3. When identifying small-areas for food policy interventions, this information helps to continuously categorize food deserts and food swamps, suggesting that Area 2 should be prioritized first because it has the lowest estimated RHFA, followed by Area 1, Area 4, and Area 3 (Table 3).

**Table 3:** Calculated RHFA and estimated RHFA in 2014

Area ID	Calculated RHFA (%)	Average of calculated RHFA in neighbouring areas (%)	Estimated RHFA(%)^a^ (95 % credible interval)
1	0	3.58	5.2 (4.1, 6.5)
2	0	2.08	4.9 (3.5, 6.6)
3	4.76	6.72	6.0 (5.0, 7.0)
4	4.76	5.31	5.3 (4.1, 6.7)

^a^ *p*_*i*4_ in Model (2) indicates the estimated RHFA in 2014

There are several limitations to this research. First, we employed a 4 km buffer for calculating RHFA. Different buffer sizes could be used depending on policy targets (e.g., improve the RHFA within a walking distance), study region characteristics (e.g., compactness), and characteristics of the local population (e.g., car ownership). The buffering size could also be altered accordingly based on food outlet types, which may be linked to the behaviours underlying travel patterns to visit specific healthy or unhealthy stores (and subtypes among them). Second, we applied the most common scheme for classifying healthy and unhealthy food outlets. The NEWPATH survey, from which food outlets were classified, measured in-store characteristics of food outlets and indicated that all non-supermarket and non-superstore outlets (e.g., full-service restaurants and pub/bars), with the exception of specialty stores (e.g., bakeries), should be categorized as unhealthy. Moreover, supermarkets/superstores are also sources of unhealthy food options. We completed additional analyses following in-store classification and counting grocery stores as both healthy and unhealthy, but results of regional and local RHFA trends (thus the identification of spatio-temporal food swamps) were similar. Additional RHFA measures based on consumer nutrition environment, for instance, shelf space devoted to healthy foods divided by the total shelf spaces devoted to healthy and unhealthy foods in accessible food outlets [55], should be considered. Lastly, we used 10 % as a threshold to define food swamps. Nevertheless, this figure could be tailored for different research contexts depending on the intervention targets for striking balance between healthy and unhealthy food access as well as evidence of the level at which RHFA impacts healthy food purchase, consumption, or health outcomes in specific study regions.

Future research should further apply this Bayesian approach in different contexts (e.g., outside Canada) to study spatio-temporal variations of the food environment accounting for transportation networks. Of particular interest is the association between changes in public transit and changes to RHFA. Future research could also analyse the association between spatio-temporal patterns of the food environment and health or socio-economic data, when available. Compared to spatial studies that analyse one time period, spatio-temporal analysis clarifies how changes in the food environment influence health outcomes (e.g., obesity), and how the food environment may be changing in tandem with increasing or decreasing socioeconomic status.

## Conclusions

This paper explores the spatio-temporal patterns of RHFA in the Region of Waterloo over 4 years, using a Bayesian spatio-temporal modelling approach. This method quantifies regional temporal trend and local spatio-temporal trends of RHFA, which are not available from traditional spatial or descriptive analyses. In particular, this study adds to the literature for investigating relative food access at a small temporal scale (based on annual RHFA changes).

Results of our study are consistent with previous findings in the Canadian context that food swamps are more prevalent than food deserts. While food deserts should be prioritized, food swamps (especially spatio-temporal food swamps) should not be overlooked by public health practitioners and policy-makers. In general, food swamps have become more prevalent during the study period, given that RHFA has decreased at the regional level and all DAs (most are food swamps in the starting year 2011) at the local level show significant decreasing trend of RHFA. Areas located at south Waterloo, north Kitchener, as well as southeast Cambridge have the steepest RHFA decreasing gradient (Fig. 3) thus are spatio-temporal food swamps and should be prioritized for interventions.

## Footnotes

1 Community nutrition environment refers to characteristics such as food outlet types and locations; consumer nutrition environment includes in-store characteristics such as food affordability, quality [56].

2 This paper follows the mRFEI approach to differentiate food deserts and food swamps.

3 The figure was derived based on Census Canada 2011.

4 In Bayesian approaches, hyperparameters are the parameters of priors. Priors assigned to hyperparameters are called hyperpriors.

5 Moran’s I is a statistical method to quantify spatial autocorrelations. A value of Moran’s I approaching 1 indicates strong positive autocorrelations.

6 +∞ means that only positive values from the normal distribution will be sampled.
